# Exploring Lifestyle and Dietary Patterns in Pregnancy and Their Impact on Health: A Comparative Analysis of Two Distinct Groups 10 Years Apart

**DOI:** 10.3390/nu16030377

**Published:** 2024-01-27

**Authors:** Andreea-Maria Mitran, Andreea Gherasim, Otilia Niță, Laura Mihalache, Lidia Iuliana Arhire, Oana Cioancă, Dumitru Gafițanu, Alina Delia Popa

**Affiliations:** 1 Faculty of Medicine, University of Medicine and Pharmacy “Grigore T. Popa”, 700115 Iasi, Romania; mitran.andreea-maria@email.umfiasi.ro; 2 Department of Internal Medicine II, Faculty of Medicine, University of Medicine and Pharmacy “Grigore T. Popa”, 700115 Iasi, Romania; otilia.nita@umfiasi.ro (O.N.); laura.mihalache@umfiasi.ro (L.M.); lidia.graur@umfiasi.ro (L.I.A.); 3 Faculty of Pharmacy, University of Medicine and Pharmacy “Grigore T. Popa”, 700115 Iasi, Romania; oana.cioanca@umfiasi.ro; 4 Department of Obstetrics and Gynecology, University of Medicine and Pharmacy “Grigore T. Popa”, 700115 Iasi, Romania; 5 Department of Nursing, Faculty of Medicine, University of Medicine and Pharmacy “Grigore T. Popa”, 700115 Iasi, Romania; alina.popa@umfiasi.ro

**Keywords:** dietary patterns, nutrients, pregnancy, gestational weight gain, nutritional epidemiology

## Abstract

The significance of dietary patterns during pregnancy is highlighted by accumulating evidence, emphasizing their pivotal role in promoting a healthy pregnancy for both the mother and the child. This study aimed to assess the current dietary patterns of pregnant women, compare the energy and nutrient intake of two distinct groups with a 10-year interval, and identify changes in dietary patterns. EPIC FFQ was applied, and its data were interpreted with the FETA program version 6 (CAMB/PQ/6/1205). By means of principal component analysis, three different food patterns were identified in each study group: vegetarian, balanced, and traditional (2013); and prudent, vegetarian, and modern (2023). Analyzing the relationship between food groups and gestational weight, we found that gestational weight gain in 2013 was positively correlated with eggs and egg dishes and milk and milk products, whereas in 2023, gestational weight gain was positively correlated with fats and oils, non-alcoholic beverages, and the modern pattern. Additionally, in 2023, pre-gestational BMI correlated positively with eggs and egg dishes. The balanced pattern emerged as a predictor for a lower likelihood of inadequate gestational weight gain in both groups. Furthermore, normal and overweight pregnant women showed a reduced likelihood of excessive gestational weight gain.

## 1. Introduction

Mounting evidence showcases that dietary patterns during pregnancy have a pivotal role in promoting a healthy pregnancy for both the mother and the child. The mother is the fetus’ only source of life-supplying nutrients. Thus, a pregnant woman’s nutritional status translates into the health of future generations. Consequently, assuring adequate nutrition encompassing both knowledge and practice in child-bearing women stands out as one of the most rewarding areas of intervention.

Concerning the mother, inadequate nutrition can alter the course of pregnancy by increasing the risk of pre-eclampsia [1], gestational diabetes [2,3], and cesarean section [4]. In terms of fetal health, inadequate nutrition may result in neural tube defects [5,6], altered neurodevelopment [7,8], intra-uterine growth restriction [9], macrosomia [10], as well as neonatal hypoglycemia [11]. Moreover, the consequences of adequate nutrition extend far beyond the immediate health of the mother and fetus. Prenatal nutrition can potentially affect the future individual’s long-term health, influencing their risk of developing non-communicable chronic diseases [12]. Lack of maternal adherence to healthy eating can increase the offspring’s risk of obesity and type 2 diabetes mellitus (T2DM) later in life [13]. Likewise, unfortunate circumstances, such as The Dutch Famine or The Great Chinese Famine, showcase the complex dynamics between prenatal and early life exposures and lifelong consequences [14,15,16]. Moreover, the mother’s nutritional literacy lays the foundation for developing healthy dietary patterns and attitudes, encouraging a positive food environment within their future families and thus influencing the health behaviors of future generations [17,18]. As such, improving dietary patterns in pregnancy has far-reaching implications for public health by fostering the health of current and future generations. 

Growing research shows that the environment plays an important role in shaping eating habits [19]. The ubiquity and accessibility of energy-dense, nutrient-poor food options, coupled with a decline in physical activity, can contribute to the adoption of suboptimal dietary patterns during pregnancy. Therefore, instilling accurate and tailored dietary practices could also help mitigate the detrimental effects of the obesogenic environment. There is a notable gap in the current body of research concerning the examination of changes in eating habits among pregnant women throughout various time intervals [20]. The absence of study is worrisome, considering the crucial significance of nutrition during pregnancy for both the health of the mother and the growth of the fetus. Current research mostly examines the effects of nutrients, specific foods, and general dietary habits on pregnancy [21,22] rather than evaluating the evolution of eating patterns in a specific population over a period of time. Prior studies have shown the significant impact of counseling in enhancing women’s understanding of the potential risks associated with GDM for both the mother and the fetus [23]. These studies have emphasized the importance of nutritional education in influencing dietary choices.

The first step in accomplishing this is gathering more information about how dietary habits have evolved as a result of the shift in the food market so that future tailored interventions can target these shortcomings. By bringing awareness to this subject, this paper intends to add to the existing literature and provide new insights to guide healthcare practices and future interventions, ultimately improving perinatal/pregnancy-related health. 

Thereupon, the aim of this study comprised three objectives: (i) to assess the dietary patterns of pregnant women in two different samples; (ii) to explore the changes in dietary patterns during pregnancy over the last decade; and (iii) to assess the relationship between gestational weight gain and dietary patterns in each of the two groups.

## 2. Materials and Methods

### 2.1. Study Design

Two separate cross-sectional studies were conducted. In 2013, a study was conducted on 400 pregnant healthy women who were randomly chosen during their last antenatal hospital visit at the Cuza Voda Obstetrics and Gynecology Hospital in Iasi. We excluded cases of stillbirth, individuals who refused to participate, individuals with obstetric pathology, or those with mental, cognitive, or other diseases that could potentially hinder their comprehension of this study’s aims or their capacity to provide correct information. A total of 410 women were recruited to participate in the research study. In total, 10 interviews were omitted from the final analysis due to drop-outs, which refers to refusals to participate after the initial stage of this study or incomplete responses. 

The second study took place in 2023 at the Elena Doamna Obstetrics and Gynecology Hospital in Iasi. The women were randomly chosen during their final hospital visit before delivery. In total, 15 pregnant women declined to participate in this study. Therefore, 251 adult women were recruited in a convenience sample at their final antenatal routine visit. The exclusion criteria for the second group included obstetrical pathologies, twin pregnancies, and denial of participation. 

### 2.2. Anthropometric Assessment and Socioeconomic and Lifestyle Data

Age, pre-gestational weight, area of residence, marital status, education, parity, and prenatal care were obtained through direct interviewing. The assessed anthropometric maternal parameters included pre-gestational BMI; this was determined from the measurements recorded by the family doctor in the patient’s file or through self-estimations if the data were not available. Additionally, the height and weight at the end of pregnancy were measured prior to birth. The total weight gain during pregnancy was calculated as the difference between the weight measured before delivery and that reported or determined at the initial visit in the first trimester by the family doctor. Research has indicated that self-reported weight could usually be precise, particularly among specific groups like pregnant women who tend to be more conscious of weight fluctuations due to continuous monitoring throughout pregnancy. When conducting epidemiological studies, which aim to find patterns and connections within a population, it is often acceptable to have some degree of variance in the accuracy of self-reported weight. This fluctuation does not have a substantial impact on the overall results drawn from this study [24,25,26].

BMI was interpreted based on the World Health Organization classification (underweight, BMI < 18.49 kg/m^2^; normal weight, BMI: 18.5–24.99 kg/m^2^; overweight, BMI: 25.00–29.99 kg/m^2^; obese ≥ 30 kg/m^2^) [27]. We applied the 2009 recommendations for gestational total weight gain classification provided by the Institute of Medicine, considering the assessment based on pre-gestational BMI guidelines: 12.5–18 kg (underweight); 11.5–16 kg (normal weight); 7–11.5 kg (overweight); 5–9 kg (obesity) [28]. Weight gain below the acceptable level set by the IOM was regarded as insufficient, while weight gain above the prescribed level was considered excessive.

### 2.3. Dietary Assessment 

Information for analyzing dietary patterns was obtained by using a previously validated food frequency questionnaire [29]. Food frequency and portion sizes were assessed using the EPIC-Norfolk Study FFQ [30], which had been previously translated and validated on the Romanian population [31]. 

### 2.4. Ethics

The current study was conducted in accordance with the Declaration of Helsinki and approved by the Ethics Committee of both Gr. T. Popa University of Medicine and Pharmacy (348/28 September 2023) and Elena Doamna Hospital of Obstetrics and Gynecology, Iasi (1373/14 February 2023). Informed consent was obtained from all subjects involved in this study.

### 2.5. Statistical Analysis

Utilizing the data and information acquired from the 2023 patient cohort, we opted for R Studio (version 1.4.1106) to construct the database and conduct the statistical tests. Within this database, we incorporated the data from the 2013 group to facilitate a comparative analysis. Descriptive analysis was used to compute the mean, median, minimum and maximum values, standard deviation, standard error, 95% confidence interval, and frequencies. Wilcoxon rank sum tests were applied to compare the parameters between the two groups (2013 vs. 2023). Spearman rho correlation was employed to identify different associations among the studied parameters, considering significant *p*-values < 0.05. Factor analysis, specifically principal component analysis, was utilized to discern dietary patterns within 13 food groups. The suitability of factor analysis to the variables was assessed using the Keyser–Meyer–Olkin (KMO) test and Bartlett’s test, with a KMO value exceeding 0.5 considered acceptable. In Bartlett’s test, significance was established at *p* < 0.05. Only components meeting the Kaiser criteria (eigenvalue above 1.3) were considered. Factor loadings above 0.25 were significant. To identify predictors of weight changes during pregnancy, we employed multinomial logistic regression and interpreted the results in terms of the odds ratio (OR).

## 3. Results

### 3.1. Population Characteristics

The median age at recruitment was 28 in the 2013 sample compared to 26 in 2023 (*p* = 0.012). Concerning pre-gestational BMI, the median value increased from 21.33 kg/m^2^ in the first sample to 24.35 kg/m^2^ in the latter (*p* < 0.001). The median gestational weight gain was 14.00 kg in 2013 and 6.70 kg in 2023 (*p* < 0.001) (Table 1). Regarding gestational age, there were no significant differences between the two groups (*p* = 0.77).

The main sociodemographic characteristics of the sample population are presented in Table 2. In 2013, 54.3% (*n* = 217) of the sample population resided in an urban environment, compared to 41% (*n* = 103) in 2023. Regarding education level, in the 2013 group, 21.5% (*n* = 86) had a middle school education; 40.5% (*n* = 162) had graduated from high school, and 34% (*n* = 136) had obtained a university degree. In comparison, in the 2023 group, 33.9% (*n* = 85) of women had a middle school education; 31.5% (*n* = 79) had completed high school, and 27.1% (*n* = 68) had obtained a university degree.

In 2013, the smoking status of women was as follows: 88.5% (*n* = 354) identified as nonsmokers; 0.5% (*n* = 2) identified as former smokers; and 11% (*n* = 44) identified as smokers. Within the 2023 group, 66.5% (*n* = 167) of women did not smoke; 10.4% (*n* = 26) had ceased smoking, and 23.1% (*n* = 58) were currently smoking. Concerning diet advice, in 2013, 42.3% (*n* = 169) of women reported receiving information about diet during pregnancy, while 57.8% (*n* = 231) denied any mention of diet advice during antenatal visits. In contrast, in the 2023 group, 14.7% (*n* = 37) reported discussing a healthy diet during pregnancy with healthcare providers, and 85.3% (*n* = 214) did not recall such advice. The proportion of pregnant women who declared receiving dietary advice was lower in 2023 (*p* < 0.001).

Regarding dietary supplements, data were recorded regarding the consumption of folate supplements (either before conception or during the initial trimester) and other nutritional supplements, predominantly multivitamins. In terms of folate supplementation, 48.3% (*n* = 193) reported taking them in 2013, while only 8% (*n* = 20) took them in 2023 (*p* < 0.001). Other nutritional supplements were used by 68.8% (*n* = 267) of women in 2013 compared to 33.5% (*n* = 84) in 2023 (*p* = 0.117).

In 2013, 82.75% (*n* = 331) had a normal BMI (between 20.0 and 24.9 kg/m^2^) as opposed to 58.19% (*n* = 142) in 2023. In the first group, 16% (*n* = 64) were overweight compared to 22.54% (*n* = 55) in the latter. Ultimately, 1.25% (*n* = 5) of women in the 2013 group had obesity compared to 19.26% (*n* = 47) in 2023 (*p* < 0.001). Concerning gestational weight gain, in 2013, it was adequate in 41.88% (*n* = 160), inadequate in 23.82% (*n* = 91), and excessive in 34.29% (*n* = 131) of cases. Comparatively, in the 2023 group, weight gain was adequate in 61% (*n* = 150), inadequate in 15.9% (*n* = 39), and excessive in 23.2% (*n* = 57) of cases (*p* < 0.001).

### 3.2. Nutrient Intake and Dietary Patterns

A thorough comparison of nutrient intake between the 2013 and 2023 groups provided valuable insights into the evolving dietary habits of the analyzed cohorts. In 2023, the consumption of total fat, monounsaturated fatty acids (MUFAs), saturated fatty acids (SFAs), cholesterol, protein, retinol, calcium, copper, phosphorus, selenium, zinc, and vitamins B2, B3, B12, C, D, and folate was lower compared to that in the previous study group. Conversely, energy intake, carbohydrates, fiber, polyunsaturated fatty acids (PUFAs), carotene, vitamin E, potassium, and sodium intake were higher in the 2023 group than in 2013. However, after adjusting for energy intake, the results remained statistically significant only for retinol, vitamin B12, and copper (Table 3, Supplementary Materials).

**Figure 1:**
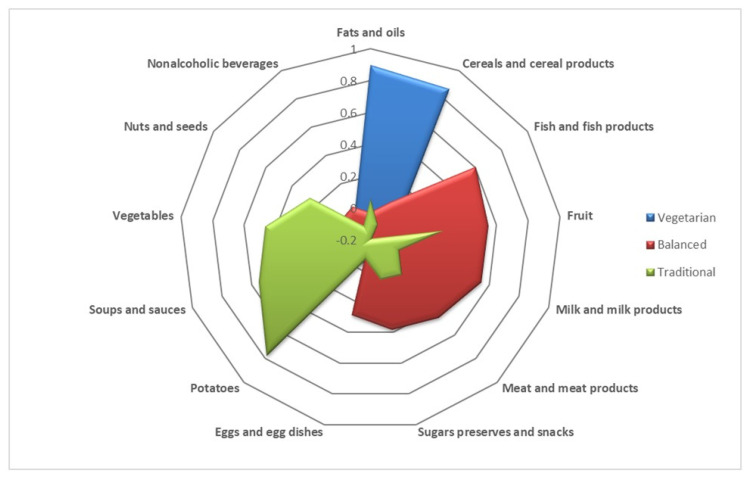
Dietary patterns in 2013 group.

We further applied factor analysis (principal component analysis) to identify dietary patterns using the 13 food groups that emerged from FETA. For the 2013 group, the KMO was 0.515. Three factors were identified that accounted for 35.3% of the overall variance. The components were named as follows: vegetarian (characterized by a high intake of fats and oils, cereals, and cereal products); balanced (characterized by a high intake of diverse foods from various dietary groups such as fish and fish products, fruits, milk and milk products, meat and meat products, sugar, preserves, snacks, and eggs and egg dishes); and traditional (characterized by a high intake of potatoes, soups, sauces, vegetables, nuts, and seeds). The factor loading values are displayed in Table 4 and Figure 1.

**Figure 2:**
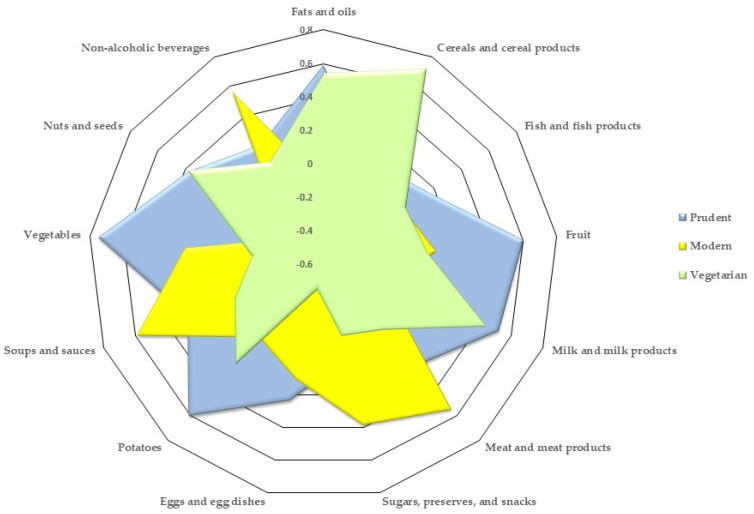
Dietary patterns in 2023 group.

The KMO value for the 2023 group was 0.562. Three components were identified, accounting for 39.6% of the overall variance. The components were labeled as follows: vegetarian (marked by a substantial intake of cereals, cereal products, nuts, and seeds); modern (marked by a significant intake of potatoes, fats, oils, soft drinks, meat, meat products, sugars, preserves, and snacks); and prudent (marked by significant consumption of vegetables, fruits, soups, sauces, milk, and dairy products). These results can be found in Table 5 and Figure 2.

### 3.3. Associations between Food Groups and Dietary Patterns with Gestational Weight Gain and Pre-Gestational BMI

The 2013 group showed a positive relationship between gestational weight gain and increased consumption of eggs and egg dishes, fish and fish products, and milk and its derivatives. Moreover, adherence to a traditional dietary pattern and a higher intake of potatoes were related to pre-gestational BMI in 2013. In the 2023 study, a positive association was observed between gestational weight gain and the consumption of fats, oils, and non-alcoholic beverages, as well as adherence to a modern dietary pattern. Furthermore, within the 2023 group, there was a positive correlation between pre-gestational BMI and increased consumption of eggs and egg dishes, as indicated in Table 6.

To investigate the relationship between weight gain during pregnancy and dietary patterns, three models were taken into consideration. In the first model, only dietary patterns were included. The second model included sociodemographic and lifestyle factors such as area of residence, maternal age, formal education, presence of dietary advice, smoking status, and parity as cofounders, and the third one added pre-gestational BMI into the analyses.

When considering adequate gestational weight gain (according to the IOM recommendation) as a comparative factor, the initial model indicated that in the 2023 group, the modern dietary pattern was associated with a significantly reduced probability of inadequate weight gain (OR = 0.317, *p* = 0.012). This relationship was preserved after adjusting for sociodemographic and lifestyle factors (OR = 0.316, *p* = 0.014). When BMI was considered as an additional cofounder, the significance of this relationship was maintained (OR =0.352, *p* = 0.03). Women with normal weight or overweight women had a reduced likelihood of experiencing excessive prenatal weight increase (OR = 0.375, *p* = 0.012 and OR = 0.286, *p* = 0.016, respectively) (Table 7).

Within the 2013 group, a significant relationship between dietary patterns and the adequacy of gestational weight gain was not found. In this group, after controlling for lifestyle and sociodemographic factors, multiparous women had a significantly increased risk of inadequate gestational weight gain (OR = 1.94, *p* = 0.035). After adjusting for pre-gestational BMI, multiparous women had a substantially higher risk of inadequate gestational weight gain (OR = 1.946, *p* = 0.035), whereas overweight women had a lower risk (OR = 0.09, *p* = 0.014). Furthermore, women with a normal pre-gestational BMI exhibited a decreased likelihood of experiencing an excessive weight increase (OR = 0.142, *p* = 0.004) (Table 7).

## 4. Discussion

In our study, we identified three dietary patterns that differed in 2023 compared to 2013 and were related to a pre-pregnancy BMI. We found an association between dietary patterns and weight gain only in the 2023 group, with women with a modern dietary pattern being less likely to have an inadequate GWG (less than recommended by the IOM) as compared to those with a vegetarian pattern.

The dietary patterns seen in observational studies showed a strong link between weight and macronutrient intake. A recent meta-analysis of 35 studies found that most observational studies conducted in pregnant women identified two types of dietary patterns: a healthy pattern, characterized by a diet high in whole grains, fruits, vegetables, and lean meat; and an unhealthy pattern, characterized by an increased intake in processed meat, fatty foods, and refined carbohydrates. Unhealthy eating has been linked to an elevated risk of premature birth or the birth of newborns with low birth weight [32]. The findings of our study revealed the presence of three main dietary patterns among each group of pregnant women, which exhibited variations over a span of 10 years. In the 2013 group, the first identified pattern was “vegetarian,” consisting of a high intake of fats and oils as well as cereals and cereal products. The next patterns identified in 2013 were “balanced,” consisting of high consumption of fish and fish products, fruits, milk and milk products; meat and meat products sugars, preserves, snacks, eggs and egg dishes, and “traditional” products, with high intakes of potatoes, soups, sauces, vegetables, nuts, and seeds.

In 2023, the “vegetarian” pattern was characterized by a high intake of cereals and cereal products, nuts, and seeds. The reduced intake of animal products, like fish and meat, suggested by the low or negative factor loadings, was a common thread among the vegetarian patterns in both the 2013 and 2023 groups, though they included different food groups. In 2023, the next two patterns were “modern,” with high intakes of potatoes, fats and oils, soft drinks, meat and meat products, sugars, preserves, and snacks and “prudent,” consisting of high consumption of vegetables, fruits, soups, sauces, milk, and dairy products.

The 2023 “modern” pattern had a lower risk of inadequate gestational weight gain, even after adjusting for pre-gestational BMI, lifestyle, and sociodemographic factors (OR = 0.352, *p* = 0.03). It is interesting to note that, at the same time, this “modern” pattern neglects main dietary guidelines and consists of high intakes of fats and oils, soft drinks, sugar, canned food, and snacks. This adds to the idea that nutritional counseling is important during pregnancy, as simply assessing gestational weight gain might not provide enough information about the nutritional status of the mother. This is particularly important, as recent research has made the case for metabolically unhealthy normal-weight individuals [33]. 

Worth noting is that only 14.7% of women received diet advice in 2023 compared to 42.3% in 2013. The decrease in nutritional education being received from professionals raises a concern regarding the factors driving food choices. When accurate information is not provided by health care practitioners, women resort to other sources of information, such as the internet. This is illustrated by recent research showing that pregnant women’s use of the internet for nutrition information has increased over recent years [18]. This highlights the need for public health interventions in this population. Through ensuring access to accurate nutritional advice, women can be empowered to make informed choices about their diet, ensuring their fetuses’ nutritional status concurrently. A study by Fallah et al. showcased a substantial increase in nutritional knowledge of pregnant women, from 3% to 31%, following nutrition education intervention [34]. Nutrition education programs have also proven to be effective in mitigating dietary practice changes during both the pregnancy [35] and the postpartum period [36]. Moreover, post-diagnosis counseling was shown to be effective in raising mothers’ awareness of how gestational diabetes mellitus could affect the fetus [23]. 

Evidence from other studies indicates the same issue regarding nutritional counseling in Romanian medical settings. Current barriers to nutrition counseling are multifaceted. The antenatal consultation guide includes advice on food safety rather than healthy eating. Given the lack of nutrition training during the undergraduate course, the little information included in the guide is difficult to interpret and put into practice by healthcare professionals [37]. Data on the national integration of nutrition counseling in healthcare settings are scarce; therefore, future research should address this matter in order to establish health policies that address this problem. Other research identified lack of time as the primary barrier faced by healthcare providers [38] followed by a lack of evidence-based nutrition education included in the medical curricula [39]. A study conducted in the UK also found that only 26% of doctors trusted their knowledge of nutrition, with 74% offering nutrition counseling less than once a month. They also reported a lack of knowledge and time as primary barriers to providing nutrition advice [40]. 

In our study, in 2013, 48.3% of women took a folate supplement, with 51.7% taking multivitamins. In contrast, in 2023, only 8% of pregnant women took folate supplements, with 33.5% taking multivitamins. In 1991, over 30 years ago, the Vitamin Study conducted by the Medical Research Council found, in a randomized interventional trial, that preconception supplementation with 4 mg of folic acid reduced the risk of a pregnancy with neural tube defects by about 72% [41]. As a result of that study, in 1998, the fortification of flour with folic acid was initiated in 78 countries. Currently, this practice has not yet been adopted in the EU. As a result, the prevalence of neural tube defects has not decreased across Europe, remaining constant at around one case per 1000 births from 1991 to the present. In comparison, with flour fortification, the prevalence of neural tube defects in Canada has decreased from 1.58 per 1000 births to 0.86 [42]. In 2021, Morris et al. attempted to determine the impact that the potential fortification of flour in 1998 would have had at a European level. They estimate that in Romania, where the estimated prevalence of neural tube defects is 0.77 per 1000 births, 746 cases could have been prevented in this manner [5]. In the absence of official national data pertaining to folic acid supplementation in Romania, owing to its over-the-counter availability, a comprehensive study conducted in 2021 analyzed a sample of 1280 pregnant women in Targu Mures. Of these, 70% did not use folic acid supplements prior to conception, and 31% did not use supplements during pregnancy either [43]. The lack of folic acid supplements associated with a low dietary intake of foods containing this nutrient, based on statistical data, should be a cause for concern. Therefore, public health interventions are imperative to increase adherence to folic acid supplementation among pregnant women in Romania, emphasizing the critical role of this nutrient in preventing neural tube defects and promoting maternal and fetal well-being.

Another alarming finding was the growing number of actively smoking pregnant women. In 2013, 11% of women were smokers, with 0.5% being ex-smokers. In 2023, the percentage of actively smoking pregnant women was 23.1%, with 10.4% being ex-smokers. In a survey of 21 centers in 17 countries, the International Child Care Practices Study discovered that a similar average of 22% of mothers smoked at the time of their child’s birth [44].

The mechanisms by which smoking affects the fetus are multiple. Directly, smoking leads to passing nicotine and carbon monoxide through the placenta into the fetal tissues. Indirectly, it affects the fetus by damaging the tissues of the placenta and affecting blood circulation in the umbilical artery. Once absorbed by the mother, due to its increased solubility in lipids, nicotine and its metabolite, cotinine, are rapidly transferred to the fetus. Moreover, analysis of nicotine and cotinine in amniotic fluid and fetal plasma suggests that more nicotine reaches the fetus than the mother. Thus, the decreased blood flow and increased vascular resistance create a state of hypoxia and malnutrition in the fetus, affecting its development [45]. Tobacco use during pregnancy can lead to numerous health defects in the fetus, including small birth weight, neurobehavioral effects, or even sudden infant death syndrome (SIDS) [46]. While the primary objective remains to motivate mothers to immediately cease smoking, if this proves unattainable, the subsequent goal is to minimize daily cigarette consumption. Detrimental consequences extend beyond the gestational period, as smoking even prior to conception correlates with an elevated risk of preterm birth [47]. Consequently, the optimal public health approach involves encouraging cessation of smoking among women of reproductive age or, at least, upon deciding to conceive. Beyond personal health, smoking has strong implications for the environment. ETS, environmental tobacco smoke caused by the mother’s smoking can pose dangers of second-hand exposure to the entire family as well as nearby individuals [48]. A paper by Rushton exploring the effects of tobacco smoke in the home highlights how this environmental health risk factor could cause children to develop SIDS, middle ear disease, and respiratory tract illnesses or exacerbate asthma [49].

The term “obesogenic environment” refers to environmental elements and conditions that promote the development of obesity in individuals. These elements include the increased availability and accessibility of unhealthy dietary options, technology advancements that foster sedentary lifestyles, and socioeconomic determinants [50]. Conversely, in our study, significant improvements were observed concerning GWG. In 2023, 61% of women had an adequate GWG compared to 41.88% in 2013. Moreover, the percentage of women who had inadequate weight gain dropped from 15.9% in 2023 compared to 23.82% in 2013. Furthermore, excessive weight gain dropped from 34.29% to 23.2%, respectively. This is a promising improvement, as studies show that GWG can have adverse effects independent of pre-pregnancy BMI [51,52]. Avoiding obesity in pregnant populations also has economic implications, as the resultant costs posed on public healthcare are considerable [53]. Children born to mothers with obesity or excessive GWG have a greater risk of being obese or overweight themselves as a result of both in utero programming and the obesogenic-prone environment in which they are born [54]. By ensuring a favorable in utero environment and addressing the current threat posed by the obesogenic environment, public health initiatives could help break the inter-generational cycle of obesity, tempering the current epidemic. 

In this context, the transition from the “balanced” and “traditional” patterns observed in 2013 to the more polarized “modern” and “prudent” patterns in 2023 stands out. This observed change in feeding patterns is in line with the research showcasing developing countries’ “nutritional transition” toward a Westernized diet [55]. In 2013, Romanian households spent over EUR 1.79 billion on catering services. By 2022, this number grew to EUR 4.87 billion [56]. Moreover, the food trade sector in Romania has become dominated by Western networks [57]. Currently, out of all deaths in Romania, 25% can be attributed to dietary factors, the third highest percentage in the EU [58]. 

With the effects of industrialization and globalization, consumption of readily packaged goods has increased. In order to increase their palatability, these products are often either high in fats and oils or in sugar. In addition, concerning the obesogenic environment, in our study, a positive correlation was observed between GWG and the food group of fats and oils (*p* = 0.002) and non-alcoholic beverages (which includes sugar-sweetened beverages (SBBs); *p* = 0.027) for the pregnant women belonging to the 2023 group. SSBs’ affordability has been strongly associated with overweight and obesity, with research reporting that an approximately 10% increase in the affordability of SSBs corresponds with 0.4 more adults who are overweight or obese per 100 citizens [59]. Excessive sugar consumption, especially the consumption of SSBs, has been inversely associated with children’s cognitive abilities [60] and with an increased risk of premature births, macrosomia, and child obesity [61]. SSBs are also high in fructose; the metabolic complications caused by high fructose intake are some of the current problems caused by an obesogenic environment. A diet high in fructose is associated with dyslipidemia, insulin resistance, and other related metabolic pathologies [62]. It should also be noted that a high-fructose diet poses risks not only for individuals who are obese but also for lean people [63].

This study also has certain limitations. The sample size in the 2023 group did not match the 2013 sample size. Other limitations include relying on self-reported data on pre-pregnancy weight as well as the relatively small sample size. Findings from this study might not be strong enough to make assumptions at a population level.

Data derived from food frequency questionnaires (FFQs) are commonly used to assess dietary patterns and have proven effective in accurately identifying maternal dietary patterns and exploring the connection between dietary patterns and health [64]. Moreover, using FFQ-derived dietary patterns is a valuable tool in nutritional epidemiology in order to measure nutritional exposure [65]. This approach has several strengths and limitations. Firstly, using principal component analysis allows for a broader view of nutritional intake patterns. In the context of assessing the habitual diet, FFQs require diminished labor and economic resources along with alleviated participant burden in contrast to other assessment methods such as weighted food journals. Thus, they reinforce greater response rates. Concurrently, certain limitations imposed by using FFQs must be acknowledged. Due to their reliance on participants’ long-term memory, recalling food intake might be inaccurate. Given their extensive nature, they necessitate longer interview sessions, potentially placing participants under strain and leading to hasty or unreflective responses. As such, the interviewer’s expertise is a key component in ensuring accurate data collection. In the interpretations of study outcomes, these shortcomings should be duly taken into account. Regarding pregnant women, PCA-derived dietary patterns produce similar information when compared to a food diary, illustrating that they preserve information about broad diet patterns [21]. Another limitation of our study was the utilization of self-reported pre-pregnancy weight when the medical data were not available. Research shows that self-reported weight accuracy is reliable, especially among pregnant women, and tolerable variation in accuracy in epidemiological research does not significantly impact general findings [66].

To the best of the authors’ knowledge, this is the first study to investigate the evolution of dietary patterns among pregnant women over a 10-year period. Other strengths of this study include the recruiting of women directly during their visit to the public hospital, a strategy that enhances the representativeness of the sample and provides an accurate reflection of the broader population of pregnant women. 

Thus, the global shift towards an obesogenic environment can be perceived in the evolution of Romanian pregnant women’s food consumption behavior. By understanding how specific population groups respond to shifts in the global environmental context, tailored interventions can be carried out to ensure success. As such, pregnant women’s nutritional status should be assessed and tackled comprehensively and holistically, not only by individual evaluation of risk factor components. One pressing matter in understanding how eating patterns are shaped is the necessity for more public health research regarding the dynamics of food markets and the prevailing obesogenic environment in Romania. The available information regarding national trends in food availability and accessibility is scarce. Therefore, to better understand the broader picture of dietary patterns, further research is warranted to address these knowledge gaps and contribute to the literature surrounding the environment and associated dietary patterns in Romania.

## 5. Conclusions

The “vegetarian” pattern in 2013, characterized by high fats and oils and cereals, shifted into a similar pattern in 2023 with an emphasis on cereals, nuts, and seeds. The “vegetarian” pattern showed consistency in certain food groups over the years. The “balanced” pattern in 2013, encompassing various food groups, shifted to a “prudent” pattern in 2023, marked by increased consumption of potatoes, fats, oils, soft drinks, meat, sugar, preservatives, and snacks. Notably, the 2023 “modern” pattern demonstrated a lower risk of inadequate gestational weight gain despite deviating from conventional dietary guidelines. This underscores the importance of nutritional counseling during pregnancy, as relying solely on gestational weight gain assessments may not provide a comprehensive understanding of maternal nutritional status, particularly considering recent research highlighting the significance of metabolic health in individuals with normal weight. Considering the entire diet over a longer period, dietary patterns provide insights into the entire diet rather than specific nutrients or food groups. From a public health perspective, this study approach allows for a comprehensive understanding of dietary habits among pregnant women, thereby enabling the development of targeted and tailored evidence-based public health interventions.

## Figures and Tables

**Table 1:** Descriptive characteristics of pregnant women.

				Percentiles		
		*n*	Median	25th	50th	75th
Age (years)	2013	400	28	23	28	31
	2023	251	26	22.5	26	30
Pre-gestational BMI (kg/m^2^)	2013	387	21.33	19.57	21.32	24.43
	2023	244	24.35	21.1	24.35	27.59
Gestational weight gain (kg)	2013	382	14	10	14	17
	2023	244	6.7	2	6.7	12.23
No. of pregnancies	2013	400	2	1	2	3
	2023	251	1	1	1	2
Gestational age (weeks)	2013	400	39	38	39	40
	2023	251	39	38	39	40

*n* = number. BMI = body mass index.

**Table 2:** Sociodemographic, anthropometry, and lifestyle factors in the 2 groups.

Parameter		2013	2023
		*n* (%)	*n* (%)
Area of residence	urban	217 (54.3)	103 (41)
	rural	183 (45.8)	148 (59)
Education level	primary school	16 (4)	19 (7,6)
	middle school	86 (21.5)	85 (33.9)
	high school	162 (40.5)	79 (31.5)
	university	136 (34)	68 (27.1)
Smoke status	no	354 (88.5)	167 (66.5)
	yes	44 (11)	58 (23.1)
	former	2 (0.5)	26 (10.4)
Diet advice	yes	169 (42.3)	37 (14.7)
	no	231 (57.8)	214 (85.3)
Folate supplements	yes	193 (48.3)	20 (8)
	no	207 (51.7)	231 (92)
Other supplements	yes	267 (68.8)	84 (33.5)
	no	133 (31.2)	167 (66.5)
BMI	normal weight	331 (82.75)	142 (58.19)
	overweight	64 (16)	55 (22.54)
	obese	5 (1.25)	47 (19.26)
Gestational weight gain	adequate	160 (41.88)	150 (61)
	inadequate	91 (23.82)	39 (15.9)
	excessive	131 (34.29)	57 (23.2)

BMI = body mass index.

**Table 3:** The average daily intake of energy and nutrients in the 2 groups.

	Year	Mean	Median	SD	*p*	*p* ^1^
Energy_kcal	2013	1835.92	1796.14	463.46	<0.001	
	2023	1992.53	1927.44	537.29		
Total CH	2013	212.83	211.20	60.24	<0.001	0.96
	2023	264.66	258.11	74.77		
Fiber	2013	115.15	114.55	36.53	<0.001	0.99
	2023	156.82	154.18	46.55		
Total fat	2013	75.54	72.29	23.41	<0.001	0.93
	2023	75.27	70.86	25.85		
MUFA	2013	28.50	26.98	10.35	0.002	0.51
	2023	26.14	24.36	10.16		
PUFA	2013	11.57	11.13	3.87	<0.001	0.62
	2023	14.54	13.46	6.14		
SFA	2013	28.24	26.82	8.74	<0.001	0.23
	2023	27.75	26.15	10.17		
Cholesterol	2013	398.76	383.88	152.91	0.001	0.51
	2023	362.96	346.04	139.94		
Protein	2013	89.00	85.88	28.58	<0.001	0.26
	2023	80.41	77.87	21.68		
Retinol	2013	2464.41	1254.56	3385.45	<0.001	0.03
	2023	755.94	361.65	975.60		
Vitamin B12	2013	11.26	7.86	11.46	<0.001	<0.001
	2023	4.94	4.01	3.78		
Folate	2013	247.39	229.64	81.83	<0.001	0.12
	2023	210.46	203.18	62.41		
Vitamin D	2013	2.79	2.56	1.40	<0.001	0.61
	2023	2.09	1.89	1.15		
Vitamin E	2013	10.79	9.91	4.88	<0.001	0.42
	2023	12.55	11.85	4.73		
Calcium	2013	859.30	843.29	193.55	<0.001	0.30
	2023	812.39	779.39	318.21		
Iron	2013	10.12	9.60	3.42	0.99	0.21
	2023	9.88	9.83	2.60		
Iodine	2013	131.39	128.72	33.04	0.81	0.40
	2023	134.33	127.60	52.42		
Magnesium	2013	248.59	240.93	64.95	0.83	0.15
	2023	251.13	239.62	70.40		
Sodium	2013	2577.43	2527.03	781.61	<0.001	0.74
	2023	2960.45	2923.41	984.57		
Selenium	2013	84.60	84.02	28.38	<0.001	0.94
	2023	77.68	76.93	21.99		
Zinc	2013	9.43	9.24	2.94	<0.001	0.65
	2023	8.34	7.92	2.45		

CH = carbohydrates; MUFA = monounsaturated fatty acids; PUFA = polyunsaturated fatty acids; SFA = saturated fatty acids; *p* ^1^ = *p* adjusted for kcal intake; SD = standard deviation.

**Table 4:** Factor loading for dietary patterns in the 2013 group.

Food Groups	Dietary Pattern		
	Vegetarian	Balanced	Traditional
Fats and oils	0.89811	−0.0264	0.05277
Cereals and cereal products	0.87099	0.0691	−0.10012
Fish and fish products	0.01653	0.6058	−0.19065
Fruit	0.11378	0.5486	0.26453
Milk and milk products	−0.03476	0.5477	−0.00698
Meat and meat products	0.07633	0.4522	0.09564
Sugars, preserves, and snacks	0.28113	0.3819	0.06046
Eggs and egg dishes	−0.05401	0.2871	−0.06703
Potatoes	−0.00119	−0.1888	0.77581
Soups and sauces	0.15452	0.1628	0.54711
Vegetables	−0.14215	0.2807	0.46199
Nuts and seeds	−0.0121	0.0207	0.25932
Non-alcoholic beverages	0.00446	0.0373	−0.10501

**Table 5:** Factor loading for dietary patterns in the 2023 study group.

Food Groups	Dietary Pattern		
	Prudent	Modern	Vegetarian
Vegetables	0.7489	0.2259	−0.17117
Fruit	0.6015	0.0773	0.0177
Soups and sauces	0.5866	0.2647	−0.03526
Milk and milk products	0.5201	−0.1906	0.43748
Fish and fish products	0.1603	−0.0155	−0.00433
Potatoes	−0.0211	0.605	0.19334
Fats and oils	−0.1141	0.5879	0.54456
Non-alcoholic beverages	0.2045	0.5634	0.08878
Meat and meat products	0.2135	0.5495	−0.07521
Sugars, preserves, and snacks	−0.0193	0.3839	−0.16185
Cereals and cereal products	0.0805	0.2474	0.71934
Eggs and egg dishes	0.2361	0.0962	−0.445
Nuts and seeds	0.3759	−0.3285	0.38559

**Table 6:** Correlations between food groups and dietary patterns with anthropometric parameters.

Parameters	2013				2023			
	Pre-Gestational BMI		Gestational Weight Gain		Pre-Gestational BMI		Gestational Weight Gain	
	r	*p*	r	*p*	r	*p*	r	*p*
Cereals and cereal products	0.052	0.309	0.098	0.057	0.125	0.051	−0.003	0.964
Eggs and egg dishes	0.043	0.397	0.138	0.007	0.133	0.038	0.031	0.630
Fats and oils	0.049	0.340	0.088	0.085	0.076	0.236	0.200	0.002
Fish and fish products	0.002	0.970	0.141	0.006	0.045	0.487	−0.044	0.492
Fruit	0.068	0.180	0.073	0.154	0.005	0.938	0.032	0.617
Meat and meat products	0.031	0.540	0.052	0.310	0.050	0.439	0.021	0.742
Milk and milk products	0.041	0.416	0.120	0.019	−0.091	0.157	0.062	0.338
Non-alcoholic beverages	−0.004	0.931	−0.026	0.616	0.001	0.987	0.142	0.027
Nuts and seeds	0.072	0.156	−0.069	0.177	0.082	0.203	0.006	0.926
Potatoes	0.102	0.046	−0.066	0.200	0.035	0.588	0.036	0.573
Soups and sauces	0.049	0.337	0.006	0.909	−0.015	0.810	−0.019	0.768
Sugars, preserves, and snacks	−0.027	0.597	0.044	0.394	−0.065	0.310	0.075	0.246
Vegetables	0.044	0.390	0.069	0.176	−0.063	0.325	0.076	0.238
Vegetarian 2013	0.039	0.439	0.086	0.092	-	-	-	-
Balanced 2013	0.94	0.066	0.180	<0.001	-	-	-	-
Traditional 2013	0.128	0.012	−0.049	0.338	-	-	-	-
Prudent 2023	-	-	-	-	−0.016	0.808	0.064	0.318
Modern 2023	-	-	-	-	0.051	0.429	0.136	0.034
Vegetarian 2023	-	-	-	-	0.069	0.284	0.018	0.777

BMI = body mass index.

**Table 7:** The relationship between dietary patterns and inadequate or excessive weight gain during pregnancy.

GWG		2013			2023	
		*p*	OR (95% CI)		*p*	OR (95% CI)
Model 1						
Inadequate	vegetarian vs. traditional pattern	0.822	0.929 (0.48–1.77)	prudent vs. vegetarian pattern	0.094	0.487 (0.21–1.13)
	balanced vs. traditional pattern	0.898	0.96 (0.51–1.80)	modern vs. vegetarian pattern	0.012	0.317 (0.12–0.77)
Excessive	vegetarian vs. traditional pattern	0.489	1.229 (0.68–2.20)	prudent vs. vegetarian pattern	0.225	0.626 (0.29–1.33)
	balanced vs. traditional pattern	0.242	1.402 (0.79–2.47)	modern vs. vegetarian pattern	0.11	0.543 (0.25–1.14)
Model 2						
Inadequate	environment	0.572	1.196 (0.64–2.22)	environment	0.752	1.136 (0.51–2.50)
	age	0.064	0.946 (0.89–1.00)	age	0.428	0.969 (0.89–1.04)
	education	0.66	0.912 (0.60–1.37)	education	0.992	0.998 (0.65–1.52)
	smoking status	0.37	1.505 (0.61–3.68)	smoking status	0.726	0.903 (0.51–1.59)
	dietary advice	0.787	1.079 (0.62–1.87)	dietary advice	0.051	2.599 (0.99–6.79)
	multiparous	0.035	1.941 (1.04–3.6)	multiparous	0.813	1.144 (0.37–3.49)
	vegetarian vs. traditional pattern	0.907	1.04 (0.53–2.02)	prudent vs. vegetarian pattern	0.062	0.432 (0.17–1.04)
	balanced vs. traditional pattern	0.676	1.151 (0.59–2.22)	modern vs. vegetarian pattern	0.014	0.316 (0.12–0.79)
Excessive	environment	0.166	0.672 (0.38–1.17)	environment	0.179	1.606 (0.80–1.13)
	age	0.495	1.017 (0.96–1.06)	age	0.55	0.98 (0.21–3.20)
	education	0.879	1.029 (0.70–1.49)	education	0.147	1.31 (0.90–1.88)
	smoking status	0.543	0.803 (0.39–1.63)	smoking status	0.984	0.995 (0.62–1.59)
	dietary advice	0.924	1.024 (0.63–1.64)	dietary advice	0.834	0.9 (0.33–2.40)
	multiparous	0.849	1.055 (0.60–1.82)	multiparous	0.895	0.936 (0.35–2.50)
	vegetarian vs. traditional pattern	0.624	1.16 (0.64–2.09)	prudent vs. vegetarian pattern	0.186	0.59 (0.27–1.29)
	balanced vs. traditional pattern	0.375	1.303 (0.72–2.33)	modern vs. vegetarian pattern	0.099	0.521 (0.24–1.13)
Model 3						
Inadequate	environment	0.542	1.22 (0.63–2.37)	environment	0.878	0.938 (0.41–2.12)
	age	0.059	0.94 (0.89–1)	age	0.267	0.956 (0.88–1.03)
	education	0.545	0.89 (0.59–1.32)	education	0.661	0.907 (0.58–1.40)
	smoking status	0.465	1.41 (0.55–3.6)	smoking status	0.795	0.926 (0.51–1.65)
	dietary advice	0.783	1.08 (0.62–1.88)	dietary advice	0.027	3.045 (1.13–8.18)
	multiparous	0.035	1.946 (1.04–3.62)	multiparous	0.774	1.18 (0.38–3.66)
	vegetarian vs. traditional pattern	0.92	1.03 (0.52–2.01)	prudent vs. vegetarian pattern	0.09	0.456 (0.18–1.13)
	balanced vs. traditional pattern	0.64	1.17 (0.60–2.28)	modern vs. vegetarian pattern	0.03	0.352 (0.13–0.90)
	normal weight vs. obesity	0.256	0.40 (0.08–1.92)	normal weight vs. obesity	0.789	0.856 (0.27–2.68)
	overweight vs. obesity	0.014	0.09 (0.01–0.62)	overweight vs. obesity	0.109	2.65 (0.80–8.73)
Excessive	environment	0.065	0.568 (0.31–1.03)	environment	0.256	1.514 (0.74–3.09)
	age	0.938	0.998 (0.94–1.05)	age	0.482	0.975 (0.90–1.04)
	education	0.762	1.061 (0.72–1.55)	education	0.18	1.291 (0.24–1.13)
	smoking status	0.569	0.80 (0.37–1.71)	smoking status	0.65	1.118 (0.69–1.81)
	dietary advice	0.815	1.060 (0.64–1.72)	dietary advice	0.9	0.938 (0.34–2.54)
	multiparous	0.981	1.006 (0.57–1.77)	multiparous	0.893	0.933 (0.33–2.57)
	vegetarian vs. traditional pattern	0.596	1.179 (0.64–2.17)	prudent vs. vegetarian pattern	0.207	0.598 (0.26–1.32)
	balanced vs. traditional pattern	0.178	1.523 (0.82–2.8)	modern vs. vegetarian pattern	0.137	0.547 (0.24–1.21)
	normal weight vs. obesity	0.004	0.142 (0.03–0.53)	normal weight vs. obesity	0.012	0.375 (0.17–0.80)
	overweight vs. obesity	0.093	0.305 (0.07–1.20)	overweight vs. obesity	0.016	0.286 (0.10–0.79)

CI = Confidence Interval; OR = odds ratio; *p* = statistical significance; GWG = gestational weight gain.

## Data Availability

Data are contained within the article and Supplementary Materials.

## Supplementary Materials

The following supporting information can be downloaded at: https://www.mdpi.com/article/10.3390/nu16030377/s1, Table S1. Descriptive characteristics of pregnant women; Table S2. The average daily intake of energy and nutrients in the 2 groups; Table S3. Relationships between dietary patterns and nutritional status during pregnancy; Table S4. The relationship between dietary patterns and inadequate or excessive weight gain during pregnancy.
