# Supplementary material for: Exploring Lifestyle and Dietary Patterns in Pregnancy and Their Impact on Health: A Comparative Analysis of Two Distinct Groups 10 Years Apart
Source: Nutrients. 2024 Jan 27;16(3):377. doi: 10.3390/nu16030377 (PMC10857126; doi:10.3390/nu16030377)
Supplement: Supplementary file 1 [file nutrients-16-00377-s001.zip › nutrients-2780579-supplementary.pdf]

Table S1. Descriptive characteristics of pregnant women.

								Percentiles		
		N	Mean	Median	SD	Minimum	Maximum	25th	50th	75th
Age (years old)	2013	400	27.58	28	5.72	15	52	23	28	31
	2023	251	26.55	26	5.44	18	40	22.5	26	30
Pre-gestational BMI (kg/m^2)	2013	387	22.37	21.33	3.96	14.69	44.98	19.57	21.32	24.43
	2023	244	25.06	24.35	5.24	15.42	45	21.1	24.35	27.59
Gestational weight gain (kg)	2013	382	14.2	14	5.15	0	30	10	14	17
	2023	244	8.34	6.7	8.47	-8.1	41	2	6.7	12.23
No of pregnancies	2013	400	2.38	2	1.71	1	13	1	2	3
	2023	251	1.73	1	0.94	1	5	1	1	2
Gestational age (weeks)	2013	400	38.57	39	1.34	31	42	38	39	40
	2023	251	38.55	39	1.39	34	42	38	39	40

N=number; SD=standard deviation.

Table S2. The average daily intake of energy and nutrients in the 2 groups

	Year	N	Mean	Median	SD	IQR	Min	Max	Percentile			p	p [1]	p [2]
									25th	50th	75th			
Energy_kcal	2013	400	1835.92	1796.14	463.46	559.33	214.05	4014.55	1543.06	1796.14	2102.38	<0.001		0.36
	2023	251	1992.53	1927.44	537.29	664.12	913.58	4183.99	1610.00	1927.44	2274.12			
Total CH	2013	400	212.83	211.20	60.24	76.16	16.00	546.62	171.06	211.20	247.23	<0.001	0.96	0.9
	2023	251	264.66	258.11	74.77	89.07	109.57	575.17	209.78	258.11	298.85			
Fiber	2013	400	115.15	114.55	36.53	48.42	0.07	291.63	90.06	114.55	138.47	<0.001	0.99	0.76
	2023	251	156.82	154.18	46.55	57.00	54.57	404.15	127.56	154.18	184.56			
Total fat	2013	400	75.54	72.29	23.41	28.89	12.65	189.53	59.60	72.29	88.49	<0.001	0.93	0.86
	2023	251	75.27	70.86	25.85	30.10	30.48	221.17	58.63	70.86	88.73			
MUFA	2013	400	28.50	26.98	10.35	11.93	3.67	89.18	21.72	26.98	33.65	0.002	0.51	0.84
	2023	251	26.14	24.36	10.16	11.35	9.03	98.85	19.47	24.36	30.82			
PUFA	2013	400	11.57	11.13	3.87	4.90	0.65	35.18	8.93	11.13	13.83	<0.001	0.62	0.09
	2023	251	14.54	13.46	6.14	5.97	3.96	65.26	10.66	13.46	16.64			
SFA	2013	400	28.24	26.82	8.74	10.38	7.27	83.39	22.34	26.82	32.72	<0.001	0.23	0.88
	2023	251	27.75	26.15	10.17	12.81	9.71	61.89	20.59	26.15	33.40			
Cholesterol	2013	400	398.76	383.88	152.91	173.55	41.02	1201.02	306.82	383.88	480.36	0.001	0.51	0.72
	2023	251	362.96	346.04	139.94	165.49	80.11	987.85	267.90	346.04	433.40			
Protein	2013	400	89.00	85.88	28.58	33.45	9.99	281.67	69.86	85.88	103.31	<0.001	0.26	0.605
	2023	251	80.41	77.87	21.68	28.62	35.22	155.61	64.94	77.87	93.56			
Carotene	2013	400	2010.96	1634.42	1248.99	1325.72	119.09	10333.09	1204.18	1634.42	2529.89	<0.001	0.67	0.69
	2023	251	3668.11	3826.53	2510.34	3731.25	106.35	18035.86	1494.32	3826.53	5225.57			
Retinol	2013	400	2464.41	1254.56	3385.45	1752.38	152.36	29242.93	488.97	1254.56	2241.35	<0.001	0.03	0.05
	2023	251	755.94	361.65	975.60	743.09	45.49	5371.35	247.72	361.65	990.81			
VitaminB1	2013	400	1.35	1.31	0.40	0.50	0.10	3.20	1.09	1.31	1.59	0.23	0.22	0.44
	2023	251	1.32	1.28	0.40	0.54	0.53	2.72	1.02	1.28	1.57			
VitaminB2	2013	400	2.15	1.87	1.02	1.17	0.51	8.89	1.47	1.87	2.64	<0.001	0.17	0.17
	2023	251	1.53	1.42	0.59	0.77	0.49	3.68	1.08	1.42	1.85			
VitaminB3	2013	400	23.01	21.79	8.95	9.85	0.53	87.77	17.23	21.79	27.07	<0.001	0.39	0.81
	2023	251	19.92	19.34	6.23	7.24	7.86	42.57	15.98	19.34	23.22			
VitaminB6	2013	400	1.92	1.87	0.56	0.76	0.21	4.49	1.52	1.87	2.28	0.34	0.43	0.37

	2023	251	1.89	1.80	0.56	0.76	0.77	4.41	1.50	1.80	2.25			
VitaminB12	2013	400	11.26	7.86	11.46	7.54	1.17	101.53	4.93	7.86	12.47	<0.001	<0.001	0.013
	2023	251	4.94	4.01	3.78	3.38	0.44	24.78	2.71	4.01	6.09			
Folate	2013	400	247.39	229.64	81.83	106.02	21.83	659.18	190.06	229.64	296.08	<0.001	0.12	0.20
	2023	251	210.46	203.18	62.41	86.17	93.13	473.65	162.05	203.18	248.22			
VitaminC	2013	400	105.70	87.02	64.80	57.04	10.92	420.48	62.72	87.02	119.75	<0.001	0.08	0.84
	2023	251	76.66	72.47	39.71	46.10	7.65	346.07	47.60	72.47	93.70			
VitaminD	2013	400	2.79	2.56	1.40	1.62	0.09	8.03	1.88	2.56	3.49	<0.001	0.61	0.28
	2023	251	2.09	1.89	1.15	1.28	0.25	7.88	1.34	1.89	2.62			
VitaminE	2013	400	10.79	9.91	4.88	4.79	0.57	48.93	7.83	9.91	12.62	<0.001	0.42	0.51
	2023	251	12.55	11.85	4.73	5.44	3.05	31.08	9.18	11.85	14.62			
Calcium	2013	400	859.30	843.29	193.55	222.35	343.19	1805.47	732.04	843.29	954.39	<0.001	0.30	0.30
	2023	251	812.39	779.39	318.21	387.40	222.24	1894.02	584.71	779.39	972.10			
Copper	2013	400	2.06	1.53	1.78	1.13	0.03	15.72	1.10	1.53	2.24	<0.001	<0.001	<0.001
	2023	251	1.31	1.19	0.60	0.45	0.47	4.43	0.98	1.19	1.43			
Iron	2013	400	10.12	9.60	3.42	3.96	0.27	27.80	7.90	9.60	11.86	0.99	0.21	0.22
	2023	251	9.88	9.83	2.60	3.46	4.93	20.10	7.96	9.83	11.41			
Iodine	2013	400	131.39	128.72	33.04	39.19	44.63	309.45	109.32	128.72	148.51	0.81	0.40	0.42
	2023	251	134.33	127.60	52.42	69.60	44.45	333.23	95.29	127.60	164.89			
Potassium	2013	400	2796.69	2739.65	706.04	827.90	464.13	5797.87	2323.00	2739.65	3150.90	0.01	0.53	0.62
	2023	251	2926.99	2813.76	836.43	1019.57	1173.86	6710.09	2346.43	2813.76	3366.00			
Magnesium	2013	400	248.59	240.93	64.95	83.08	37.92	473.04	202.17	240.93	285.25	0.83	0.15	0.88
	2023	251	251.13	239.62	70.40	85.18	107.68	578.37	201.76	239.62	286.93			
Sodium	2013	400	2577.43	2527.03	781.61	943.66	167.50	7045.86	2073.21	2527.03	3016.88	<0.001	0.74	0.66
	2023	251	2960.45	2923.41	984.57	1401.12	1069.11	6199.50	2237.04	2923.41	3638.15			
Phosphorus	2013	400	1318.60	1292.61	337.31	422.84	281.59	3046.19	1088.51	1292.61	1511.35	0.01	0.73	0.77
	2023	251	1266.76	1219.55	368.54	477.18	513.47	2614.82	994.52	1219.55	1471.71			
Selenium	2013	400	84.60	84.02	28.38	35.18	3.26	224.30	65.13	84.02	100.31	<0.001	0.94	0.73
	2023	251	77.68	76.93	21.99	26.53	28.03	187.31	63.16	76.93	89.69			
Zinc	2013	400	9.43	9.24	2.94	3.73	1.25	24.62	7.40	9.24	11.12	<0.001	0.65	0.85
	2023	251	8.34	7.92	2.45	3.18	3.99	18.30	6.52	7.92	9.69			

CH=carbohydrates; MUFA=monounsaturated fatty acids; PUFA=polyunsaturated fatty acids; SFA=saturated fatty acids; p^1=p adjusted for BMI; p^2=p adjusted for kcal and BMI

Table S3. Relationships between dietary patterns and nutritional status during pregnancy.

Parameters	2013				2023			
	Pre-gestational BMI		Gestational weight gain		Pre-gestational BMI		Gestational weight gain	
	r	p	r	p	r	p	r	p
Cereals and cereal products	0.052	0.309	0.098	0.057	0.125	0.051	-0.003	0.964
Eggs and egg dishes	0.043	0.397	0.138	0.007	0.133	0.038	0.031	0.630
Fats and oils	0.049	0.340	0.088	0.085	0.076	0.236	0.200	0.002
Fish and fish products	0.002	0.970	0.141	0.006	0.045	0.487	-0.044	0.492
Fruit	0.068	0.180	0.073	0.154	0.005	0.938	0.032	0.617
Meat and meat products	0.031	0.540	0.052	0.310	0.050	0.439	0.021	0.742
Milk and milk products	0.041	0.416	0.120	0.019	-0.091	0.157	0.062	0.338
Non-alcoholic beverages	-0.004	0.931	-0.026	0.616	0.001	0.987	0.142	0.027
Nuts and seeds	0.072	0.156	-0.069	0.177	0.082	0.203	0.006	0.926
Potatoes	0.102	0.46	-0.066	0.200	0.035	0.588	0.036	0.573
Soups and sauces	0.049	0.337	0.006	0.909	-0.015	0.810	-0.019	0.768
Sugars preserves and snacks	-0.027	0.597	0.044	0.394	-0.065	0.310	0.075	0.246
Vegetables	0.044	0.390	0.069	0.176	-0.063	0.325	0.076	0.238
Vegetarian 2013	0.039	0.439	0.086	0.092	-	-	-	-
Diverse 2013	0.94	0.066	0.180	< 0.001	-	-	-	-
Traditional 2013	0.128	0.012	-0.049	0.338	-	-	-	-
Wholesome2023	-	-	-	-	-0.016	0.808	0.064	0.318
Modern2023	-	-	-	-	0.051	0.429	0.136	0.034
Vegetarian 2023	-	-	-	-	0.069	0.284	0.018	0.777

Table S4. The relationship between dietary patterns and inadequate or excessive weight gain during pregnancy.

	GWG	2013			2023	
		p	OR (95% CI)		p	OR (95% CI)
	Model 1					
inadequate	vegetarian vs traditional pattern	0.822	0.929 (0.48-1.77)	prudent vs vegetarian pattern	0.094	0.487 (0.21-1.13)
	balanced vs traditional pattern	0.898	0.96 (0.51-1.80)	modern vs vegetarian pattern	0.012	0.317 (0.12-0.77)
excessive	vegetarian vs traditional pattern	0.489	1.229 (0.68-2.20)	prudent vs vegetarian pattern	0.225	0.626 (0.29-1.33)
	balanced vs traditional pattern	0.242	1.402 (0.79-2.47)	modern vs vegetarian pattern	0.11	0.543 (0.25-1.14)
	Model 2					
inadequate	environment	0.572	1.196 (0.64-2.22)	environment	0.752	1.136 (0.51-2.50)
	age	0.064	0.946 (0.89-1.00)	age	0.428	0.969 (0.89-1.04)
	education	0.66	0.912 (0.60-1.37)	education	0.992	0.998 (0.65-1.52)
	smoke-status	0.37	1.505 (0.61-3.68)	smoke-status	0.726	0.903 (0.51-1.59)
	dietary advice	0.787	1.079 (0.62-1.87)	dietary advice	0.051	2.599 (0.99-6.79)
	multiparous	0.035	1.941 (1.04-3.6)	multiparous	0.813	1.144 (0.37-3.49)
	vegetarian vs traditional pattern	0.907	1.04 (0.53-2.02)	prudent vs vegetarian pattern	0.062	0.432 (0.17-1.04)
	balanced vs traditional pattern	0.676	1.151 (0.59-2.22)	modern vs vegetarian pattern	0.014	0.316 (0.12-0.79)
excessive	environment	0.166	0.672 (0.38-1.17)	environment	0.179	1.606 (0.80-1.13)
	age	0.495	1.017 (0.96-1.06)	age	0.55	0.98 (0.21-3.20)
	education	0.879	1.029 (0.70-1.49)	education	0.147	1.31 (0.90-1.88)
	smoke-status	0.543	0.803 (0.39-1.63)	smoke-status	0.984	0.995 (0.62-1.59)
	dietary advice	0.924	1.024 (0.63-1.64)	dietary advice	0.834	0.9 (0.33-2.40)
	multiparous	0.849	1.055 (0.60-1.82)	multiparous	0.895	0.936 (0.35-2.50)
	vegetarian vs traditional pattern	0.624	1.16 (0.64-2.09)	prudent vs vegetarian pattern	0.186	0.59 (0.27-1.29)
	balanced vs traditional pattern	0.375	1.303 (0.72-2.33)	modern vs vegetarian pattern	0.099	0.521 (0.24-1.13)
	Model 3					
inadequate	environment	0.542	1.22 (0.63-2.37)	environment	0.878	0.938 (0.41-2.12)
	age	0.059	0.94 (0.89-1)	age	0.267	0.956 (0.88-1.03)
	education	0.545	0.89 (0.59-1.32)	education	0.661	0.907 (0.58-1.40)
	smoke-status	0.465	1.41 (0.55-3.6)	smoke-status	0.795	0.926 (0.51-1.65)
	dietary advice	0.783	1.08 (0.62-1.88)	dietary advice	0.027	3.045 (1.13-8.18)

	multiparous	0.035	1.946 (1.04-3.62)	multiparous	0.774	1.18 (0.38-3.66)
	vegetarian vs traditional pattern	0.92	1.03 (0.52-2.01)	prudent vs vegetarian pattern	0.09	0.456 (0.18-1.13)
	balanced vs traditional pattern	0.64	1.17 (0.60-2.28)	modern vs vegetarian pattern	0.03	0.352 (0.13-0.90)
	normal weight vs obesity	0.256	0.40 (0.08-1.92)	normal weight vs obesity	0.789	0.856 (0.27-2.68)
	over weight vs obesity	0.014	0.09 (0.01-0.62)	over weight vs obesity	0.109	2.65 (0.80-8.73)
excessive	environment	0.065	0.568 (0.31-1.03)	environment	0.256	1.514 (0.74-3.09)
	age	0.938	0.998 (0.94-1.05)	age	0.482	0.975 (0.90-1.04)
	education	0.762	1.061 (0.72-1.55)	education	0.18	1.291 (0.24-1.13)
	smoke-status	0.569	0.80 (0.37-1.71)	smoke-status	0.65	1.118 (0.69-1.81)
	dietary advice	0.815	1.060 (0.64-1.72)	dietary advice	0.9	0.938 (0.34-2.54)
	multiparous	0.981	1.006 (0.57-1.77)	multiparous	0.893	0.933 (0.33-2.57)
	vegetarian vs traditional pattern	0.596	1.179 (0.64-2.17)	prudent vs vegetarian pattern	0.207	0.598 (0.26-1.32)
	balanced vs traditional pattern	0.178	1.523 (0.82-2.8)	modern vs vegetarian pattern	0.137	0.547 (0.24-1.21)
	normal weight vs obesity	0.004	0.142 (0.03-0.53)	normal weight vs obesity	0.012	0.375 (0.17-0.80)
	overweight vs obesity	0.093	0.305 (0.07-1.20)	overweight vs obesity	0.016	0.286 (0.10-0.79)

CI = Confidence Interval ; OR = odds ratio; p-statistical significance; GWG = gestational weight gain
